# Girls and Young Women in Community Sport: A South Australian Perspective

**DOI:** 10.3389/fspor.2021.803487

**Published:** 2022-01-14

**Authors:** Murray Drummond, Claire Drummond, Sam Elliott, Ivanka Prichard, Jamie-Lee Pennesi, Lucy K. Lewis, Christopher Bailey, Nadia Bevan

**Affiliations:** ^1^College of Education, Psychology and Social Work, SHAPE Research Centre, Flinders University, Adelaide, SA, Australia; ^2^College of Nursing and Health Sciences, Caring Futures Institute, Flinders University, Adelaide, SA, Australia; ^3^Department of Psychological and Brain Sciences, University of Louisville, Louisville, KY, United States; ^4^Faculty of Arts, School of Social Sciences, Monash University, Melbourne, VI, Australia

**Keywords:** girls, women, community sport, South Australia, qualitative and quantitative research

## Abstract

Girls' and young women's engagement and disengagement in physical activity has been well documented in Western culture. Sport plays a pivotal role in the development of behaviours that promote physical activity, particularly through commitment to team and individual goal attainment, socialisation, and feelings of belonging and self-identity. Community sport in Australia is the dominant pathway into state, national, and elite international competition. The importance of community sport in the lives of girls and young women cannot be overstated, irrespective of individual long-term sporting goals. Indeed, the dropout rate of girls in sports, like many other western cultures is significant and is certainly disproportionate to the numbers of boys who drop out. The present study aims to examine the intrapersonal, interpersonal, and environmental influences on community sporting pathways for girls and young women. Using a mixed-methods design, we include survey data from 2,189 high-school students (aged 12–18 years) and focus group and individual interview data from a subset of 37 high-school students, parents, and teachers, across metropolitan Adelaide, South Australia. The study included an examination of sporting practises and insights of male sport participants from the same age groups to juxtapose the findings and provide a more comprehensive understanding of girls' and young women's community sporting involvement. Parents and teachers were also included within the participant cohort to provide a comprehensive perspective. The results highlight the challenges that girls face with respect to engagement and disengagement in sport and particular points throughout their adolescent years. Recommendations are provided to help mitigate potential attrition of girls in sport in the future.

## Introduction

It is well known that participation in organised sport is associated with wide ranging social, psychological and physical health-related benefits (Harlow et al., 2018; Reece et al., 2020). Despite this, children and adolescents regularly withdraw from organised sport due to a range of intrapersonal, interpersonal, and environmental factors (Eime et al., 2020). Furthermore, in many Western countries, girls are more likely to discontinue organised sport throughout their adolescence in comparison to boys (Zarrett et al., 2020; Carcamo et al., 2021). A recent study in the USA investigating girls complete exit from sports participation (as opposed to girls disengaging from one sport but taking up another) found that not only are dropout rates higher among girls, but girls are more likely to never engage, or currently be playing sports in comparison to boys (Zarrett et al., 2020). Australian studies have shown that even though some girls might sample sport during childhood through modified and entry level sport programs, fewer than 25% successfully transition into organised club sport beyond the formative years, ages 6–12 (Eime et al., 2019). Gender differences are even more pronounced when looking at the continuation of sport throughout childhood and beyond with boys more likely to stay engaged, while girls will often disengage due to issues such as the competitive element of sport (Harlow et al., 2018; Reece et al., 2020). Given that one of Sport Australia's main targets released in their “Sports 2030” report is to engage more people of all ages in sport and physical activity throughout every stage of their life (Department of Health, 2018), it is important that current barriers are identified and that solutions are found in order to attract and retain girls in sport.

One key barrier that appears to remain for many girls and young women is attitudinal inequalities at the family level (e.g., discouragement by parents and relatives; Cooky et al., 2016). These attitudes serve to maintain the view that “girls” and “sport” are incompatible. Attitudinal inequalities are further compounded by broader stereotypes (e.g., the “frailty” myth), the maintenance and perpetuation of poor perceptions of body image in Physical Education settings, and a lack of familial support for both sport and academic study (Cooky et al., 2016). Another barrier surrounds the conflicting gendered performances that girls must learn to navigate if they do decide to pursue sport. Krane et al. (2014) suggest that girls can experiment with how they perform gender within sport if there are strong friendships and social support available to act as a “safety net” for negotiating gender. However, the capacity to explore the spectrum of gendered performance is inhibited by “peer-policing,” whereby girls are actively involved in reinforcing sport as contexts for judgement and approval (or disapproval) of gendered behaviour (Metcalfe, 2018). One consequence is that girls may hold back effort and downplay accomplishments in order to reinforce humility and “proper femininity” (Clark and Paechter, 2007).

The consequences of these deeply seeded sociocultural barriers are self-evident in the broader literature. In comparison to boys, research has shown that girls feel more self-conscious about their appearance and self-perceived image (Slater and Tiggemann, 2010). Girls also experience higher degrees of teasing from sporting experiences and consequently report higher levels of self-objectification and body image concerns (Slater and Tiggemann, 2011). Furthermore, they are more likely to be laughed at because of their appearance and perceived competence and be called names associated with body size and weight (Slater and Tiggemann, 2011). These perspectives are worthy of further inquiry given that contemporary sport opportunities have challenged the dominant attitudes and conversations surrounding girls sport in Australia (Elliott et al., 2020).

Developing effective pathways into and across sport, from general participation to more intensified involvement, is key to attracting and retaining girls in sport but little is known about sporting pathways for girls and young females. Indeed, sporting models have largely followed the pyramid approach that sees many athletes and participants funnelled into the sport with the most resilient and dedicated being left at the end, or in this instance, at the top of the pyramid (Green, 2005). Further, the typical sport participation model adhered to in the social and scientific community is one where children diversify their involvement in sport during the formative years, only to decrease their involvement to perhaps just one sport between 12 and 15 years (Côté and Vierimaa, 2014). Understanding the factors that impact sporting pathway choice and accessibility for adolescents and young adults in school and community sport programs will assist in improving sporting pathways from grassroots to specialised professional sport. It will also assist in shaping policies that support introduction, transition across, and retention in sport. Such scientific inquiries also support recent calls for more comprehensive examinations about the interconnectedness organised youth sport systems that are comprised of parents, siblings, peers, coaches, organisations, communities, and societies (Dorsch et al., 2020). Therefore the main aim of this study was to examine the intrapersonal, interpersonal, and environmental influences on community sporting pathways for girls and young women in Adelaide, South Australia. However, it was also designed to:

1. Explore current sporting pathways and forms of engagement for girls and young women involved in international, national, state and community-based sports in comparison to males;
2. Undertake a metropolitan-wide survey and follow-up focus groups with girls, young women and key stakeholders;
3. Understand the facilitators and barriers to girls and youth sporting engagement across all levels of sport;
4. Understand the reasons, motives and contextual factors leading to adolescents and young adults' participation in organised sport;
5. Explore the aspects of participation that help to determine what keeps adolescents and young adults involved in sport;
6. Develop recommendations for the South Australian Government Office for Recreation, Sport and Racing to assist the planning and development of strategies in improving female sporting participation and retention in South Australia.

A socio-ecological based model underpinned the research, which was ideal given that according to Mehtälä et al. (2014, P.2):

It has been suggested that a comprehensive approach, such as that offered by the socio-ecological model, is essential for examining the multiple level factors that might be determinants of physical activity (PA). The model helps us to identify opportunities to promote PA by recognising the individual (e.g., sex, beliefs, and attitudes), behavioural (sedentary and active time), and social environmental (family, teachers, peers) and physical environmental (e.g. availability of PA equipment and facilities) factors that may influence one's ability to be sufficiently physically active.

Using a mixed-methods design, we include both survey data from high-school students and focus group and individual interview data from a subset of high-school students, parents and teachers, across metropolitan Adelaide, South Australia. The study included an examination of sporting practises and insights of male sport participants from the same age groups to juxtapose the findings and provide a more comprehensive understanding of girls' and young women's community sporting involvement. Parents and teachers were also included within the participant cohort to provide a comprehensive perspective.

## Methodology

### Study Design

A mixed-methods sequential explanatory design was utilised in order for us to gain a comprehensive understanding of the issues facing girls and young women with respect to sport involvement through a broad and extensive survey. Qualitive interviews and focus groups then allowed us to gain a deeper understanding of the issues that were raised in the analyses of the surveys. Ethical approval was gained from the university institution, the Department of Catholic Education, and the Department for Education in Adelaide, South Australia. This study was completed in two distinct phases: Phase 1 consisted of a cross-sectional survey, and Phase 2 consisted of a series of focus groups and individual interviews. Data were collected between 2017 and 2020.

### Participant Recruitment and Procedure

For Phase 1 (survey), a random selection of 15 high-schools from different indices of community socio-educational advantage in metropolitan Adelaide, South Australia were invited to participate in the study. Nine schools agreed to participate and were included in the final sample. Following consent from school Principals, information packs were distributed to the parents and caregivers of students in years 8, 10, and 12 at participating schools. These packs included study information to ensure consenting participants were ethically informed. Parents and caregivers were provided with an option to opt-out of the study for their child/children. A face-to-face meeting was subsequently organised with the participating school for a member of the research team to distribute a paper-based questionnaire to participating year levels, entitled the Young People (Girls) in Sport Survey. Students were given the option to opt-out of completing the questionnaire at this point.

For Phase 2 (focus groups and individual interviews), participants were recruited in two ways. First, parents and teachers were invited to participate in focus groups at the time they were provided with information about the study, prior to Phase 1. If willing to be contacted to participate in focus groups at a later date, they were asked to provide their name, contact information, and preferred contact method, so that they could be contacted by the researcher at a later date. Second, at the end of the Phase 1 survey, all high-school students were invited to participate in focus groups, and a subset of high-school students selected at random were invited to participate in individual interviews. If willing to be contacted to participate at a later date, students were asked to provide their name, date of birth, email address, as well as their school and class teacher, so that they could be contacted by the researcher at a later date. Interested parents, teachers, and high-school students were then contacted by email to schedule focus groups and individual interviews. Focus group and individual interview questions and topics were inductively developed from the key themes identified from the Phase 1 survey results and relevant literature. An interview guide was developed and used as a means of consistency to allow for the data to be collected in a systematic manner. Participants involved in focus groups were separated into distinct groups of teachers, parents, or students in year 8, 10 or 12.

There were 4 focus groups conducted with the children year levels (*n* = 21), while there were two focus groups with teachers (*n* = 11). There were five individual interviews conducted with parents (*n* = 5) as it was difficult in access the parents at the same time for focus groups. In terms of qualitative data, this is a substantial number of participants.

Qualitative data collection took place at schools during lunch time breaks (for students and teachers) and at workplaces or places of residence (for parents). Use of focus groups and individual interviews allowed for the participants to share their stories and experiences and to express themselves without forcing them to use predetermined answers (Eliasson and Johansson, 2021). To create trustworthy qualitative data, a friendly atmosphere was established by ensuring that the participants felt safe and well informed through use of constant feedback, and the creation of a nurturing environment where all voices were acknowledged (Alderson, 2004).

### Phase 1 Variables

The survey consisted of demographic items (e.g., date of birth, ethnicity), questions about sporting activities, and six validated questionnaires measuring variables of interest. The questionnaire took between 30–45 mins to complete dependent on the year level of the participant.

#### Demographics

All participants were asked to indicate their gender, ethnicity, age, and whether they identify with having any disability or impairment.

#### Organised Sport

To gain a comprehensive understanding of the sporting activities undertaken by participants, all participants were asked to indicate if they were currently involved (or had been involved in the last 12 months) in organised sport, and if so, the number of organised sports played, types of organised sports played, time spent training, and time spent competing. Organised sport was defined as team sports such as, but not limited to, Australian football, netball or hockey, or individual sports such as athletics, triathlon, swimming or rowing.

#### Perceptions of Parental Involvement in Sport

Perceptions of parental involvement in sport was measured using 10 items from the Directive Behaviour subscale and 4 items from the Praise and Understanding subscale from the Parental Involvement in Sport Questionnaire (PISQ; Lee and MacLean, 1997). Items were adapted to allow for the assessment of a wide range of sports (i.e., “game” changed to “game/match/sports event”, “playing” changed to “competing”, “hockey” changed to “sport”). Items were scored on the frequency with which each behaviour is (a) exhibited by, and (b) desired of their parents, on 5-point scales ranging from 1 (*always*) to 5 (*never*). Satisfaction or dissatisfaction with each behaviour was calculated as the discrepancy between perceived and desired behaviour, with discrepancies ranging from −4 to +4. Scores were reported separately for mum/caregiver 1 and for dad/caregiver 2.

#### Sport Motivation

Reasons (or motivations) for sport participation was measured by the 18-item Sport Motivation Scale-II (SMS-II; Pelletier et al., 2013). The SMS-II includes six 3-item motivation subscales including: intrinsic, integrated, identified, introjected, external, and amotivated. Items were scored on 7-point scales ranging from 1 (*does not correspond at all*) to 7 (*corresponds exactly*), with higher scores indicating higher endorsement of that item/type of motivation.

#### Resilience

Resilience (or the ability to bounce back) was measured using the 6-item Brief Resilience Scale (BRS; Smith et al., 2008). Items were scored on 5-point Likert scales ranging from 1 (*strongly agree*) to 5 (*strongly disagree*). Scores were reverse coded so that higher scores indicated higher resilience.

#### Self-Esteem

General self-esteem was measured using the 10-item Rosenberg Self-Esteem Scale (RSE; Rosenberg, 1965). Items were scored on 4-point Likert scales ranging from 1 (*strongly agree*) to 4 (*strongly disagree*). Scores were reverse coded so that higher scores indicated higher self-esteem.

#### Body Appreciation

Body appreciation—that is, the acceptance, respect, and attention towards bodily needs and favourable opinions towards one's body—was measured using the 10-item Body Appreciation Scale-2 (BAS-2; Tylka and Wood-Barcalow, 2015). Items were scored on 5-point Likert scales ranging from 1 (*never*) to 5 (*always*), with higher scores indicating higher body appreciation.

### Data Management and Analyses

Phase 1 (survey): Data analysis for the quantitative phase was conducted using the Statistical Package for the Social Sciences (SPSS) software 25.0 (SPSS, 2017). Data were screened for normality and no imputations were used for missing data (Tabachnick and Fidell, 2007; Enders, 2010). Descriptive statistics were used to describe the sample (i.e., means, percentages), and inferential statistics were used to establish differences between groups (e.g., by gender, age). Comparisons were provided for the following demographic characteristics: involvement in organised sport (i.e., current, past 12 months, more than 12 months ago, never), number of organised sports played, types of organised sports played, time spent training, time spent competing, and sport motivation. Comparisons were split either by group (i.e., whole sample, males, females), year level (year 8, 10, 12), or by gender (i.e., males, females).

Phase 2 (focus groups and individual interviews): A thematic analysis approach was used to explore information central to the research aim (Braun and Clarke, 2006; Braun et al., 2017). A reflective journal was used to record field notes and audio recordings were transcribed verbatim. One research assistant reviewed transcripts for accuracy against the original audio recordings (Braun and Clarke, 2006; Braun et al., 2017). Audio-recordings and transcripts were read and re-read to build familiarity with the data. Two qualitative researchers coded data manually using open coding technique. Assigned initial codes were collated to condense the data into sub-categories and categories. The researchers met on a regular basis to review codes, sub-categories, and categories in order to increase the trustworthiness of the findings (Braun and Clarke, 2006; Braun et al., 2017).

## Phase 1 Results

### Phase 1 Participant Characteristics

In Phase 1 (survey), participants were 2,189 high-school students (983 females, 1,164 males, 38 did not specify) with a mean age of 15 years (*SD* = 1.39 years). Participants were from year 8 (44.4%), year 10 (44.2%), and year 12 (11.4%). The majority of participants reported that they were Caucasian (61.5%), followed by Asian (13.4%), Indian (5.8%), Aboriginal/Torres Strait Islander (3.4%), African (1.2%), and the remainder reported as Other (11.1%). Most participants (88.2%) identified as not having a disability or impairment. Table 1 provides a summary of participant characteristics for Phase 1 for the whole sample and by gender.

**Table 1:** Summary of participant characteristics for phase 1.

	**All**	**Females**	**Males**
	**(*N* = 2,189)**	**(*N* = 983)**	**(*N* = 11,64)**
	**Mean (SD)**	**Mean (SD)**	**Mean (SD)**
*Age*	15.03 (1.39)	15.01 (1.40)	15.05 (1.38)
	***N*** **(%)**	***N*** **(%)**	***N*** **(%)**
*Year level*			
Year 8	972 (44.4%)	432 (43.9%)	518 (44.5%)
Year 10	968 (44.2%)	431 (43.8%)	519 (44.6%)
Year 12	249 (11.4%)	120 (12.2%)	127 (10.9%)
*Ethnicity*			
Aboriginal/Torres Strait Islander	75 (3.4%)	36 (3.7%)	35 (3.0%)
Caucasian	1347 (61.5%)	617 (62.8%)	707 (60.7%)
African	27 (1.2%)	9 (0.9%)	18 (1.5%)
Asian	293 (13.4%)	113 (11.5%)	177 (15.2%)
Indian	127 (5.8%)	58 (5.9%)	68 (5.8%)
Other	243 (11.1%)	109 (11.1%)	128 (11.0%)
*Disability Impairment*			
Yes	207 (9.5%)	81 (8.2%)	116 (10.0%)
No	1931 (88.2%)	883 (89.8%)	1018 (87.5%)

*SD, Standard Deviation*.

## Participation in Organised Sport

Approximately half of the participants (52.0%) reported being currently involved in an organised sport with a further 14.8% having played sport in the past 12 months. Some participants reported being involved in an organised sport more than 12 months ago (17.6%), and 14.3% reported never being involved in an organised sport. A similar pattern was found across genders, with 63.7% of females overall and 70.1% of males overall reporting being involved in an organised sport currently or in the past 12 months. In addition, 16.4% of females and 11.8% of males reported never being involved in an organised sport. Table 2 provides a further breakdown of involvement across groups.

**Table 2:** Summary of involvement in organised sport.

	**All**	**Females**	**Males**
	**(*N* = 2,189)**	**(*N* = 983)**	**(*N* = 1,164)**
	***N* (%)**	***N* (%)**	***N* (%)**
*Sport involvement*			
Current	1138 (52.0%)	480 (48.8%)	642 (55.2%)
Past 12 months	324 (14.8%)	146 (14.9%)	174 (14.9%)
More than 12 months ago	385 (17.6%)	186 (18.9%)	194 (16.7%)
Never	314 (14.3%)	161 (16.4%)	137 (11.8%)
Not answered	28 (1.3%)	10 (1.0%)	17 (1.5%)

The top five sports played overall were Soccer (also referred to as Football; 20.1%), Australian Football (AFL; 19.2%), Netball (18.4%), Volleyball (17.5%), and Basketball (16.2%). Although, it should be noted that one school involved in the study was heavily focused on volleyball and this may have influenced these percentages. When examined by gender, different preferences were evident. For females, the top five most played sports were Netball (41.5%), Volleyball (21.9%), AFL (13.1%), Dancing (12.6%), and Soccer (10.1%). Whereas the top five sports played by males were Soccer (or Football; 27.8%), AFL (21.8%), Basketball (21.1%), Cricket (18.3%), and Volleyball (14.6%).

The most common level of sport participation was at the club/community level (40.2%), followed by school (11.2%), state (9.4%), national (3.9%) and international (1.6%). There was no significant difference between males and females in the proportion of participants that played sports at different levels, χ^2^ (5, *N* = 1,440) = 5.398, *p* = 0.369.

## Participation in Organised Sport by Gender and Year Level

Of the 1,461 participants who reported being involved in an organised sport currently, or in the past 12 months, 47.1% played 1 sport, 32.7% played 2 sports, and 7.7% played 3 or more sports. Overall, there was a significant difference in the proportion of males and females who played a different number of sports, χ^2^ (3, *N* = 2,147) = 16.09, *p* = 0.001. From Table 3, it can be seen that overall males (35.5%) were more likely to play at least one sport than females (29.7%) and females (36.3%) were more likely to not play any sport in comparison to males (29.7%).

**Table 3:** Summary of the number of organised sports (current/past 12 months only) by gender and year level.

	**All**	**Females**	**Males**
	**(*N* = 2,147)**	**(*N* = 983)**	**(*N* = 1,164)**
	***N* (%)**	***N* (%)**	***N* (%)**
*Number of organised sports*			
*N* = 0	703 (32.7%)	357 (36.3%)	346 (29.7%)
*N* = 1	705 (32.8%)	292 (29.7%)	413 (35.5%)
*N* = 2	476 (22.2%)	203 (20.7%)	273 (23.5%)
*N* = 3 or more	263 (12.2%)	131 (13.3%)	132 (11.3%)
	**Year 8**	**Year 10**	**Year 12**
	***N*** **(%)**	***N*** **(%)**	***N*** **(%)**
*Females (N = 983)*	*N* = 432	*N* = 431	*N* = 120
*N* = 0	114 (26.4%)	174 (30.1%)	69 (57.5%)
*N* = 1	118 (27.3%)	143 (33.2%)	31 (25.8%)
*N* = 2	107 (25.5%)	78 (18.1%)	15 (12.5%)
*N* = 3 or more sports	54 (20.8%)	36 (8.4%)	5 (4.2%)
*Males (N = 1164)*	*N* = 518	*N* = 519	*N* = 127
*N* = 0	124 (23.9%)	156 (30.1%)	66 (52.0%)
*N* = 1	179 (34.6%)	197 (38.0%)	37 (29.1%)
*N* = 2	139 (26.8%)	117 (22.5%)	17 (13.4%)
*N* = 3 or more sports	76 (14.7%)	49 (9.4%)	4 (5.5%)

Across the different year levels, there was a significant difference in the percentage of participants who played organised sports, χ^2^ (6, *N* = 2,189) = 117.20, *p* < 0.001. Only 25.4% of year 8's did not play any sport, compared to 54.2% of year 12's who did not play any sport. When examined by gender, in year 8 and year 10 there was a significant difference in the proportion of males and females and the number of sports played (see Table 3). For year 8's, while there was little difference in the proportion of males (23.9%) vs females (26.4%) who did not play any sport, males appeared significantly more likely to play at least 1 sport (34.6%) in comparison to females (27.3%), and males were less likely to play 3 or more sports (14.7%) than females (20.8%), χ^2^ (3, *N* = 950) = 9.80, *p* = 0.020. This pattern was different for year 10's, whereby 40.4% of females no longer played any sport in comparison to 30.1% of males, males were more likely than females to play 1 sport (38.0 vs 33.2% respectively), and there was little difference in the proportion of males and females who play 3 or more sports (9.4 vs 8.4% respectively). Overall, this pattern of differences in proportions was significant, χ^2^ (3, *N* = 950) = 11.29, *p* = 0.010. For year 12's, there was no difference in the proportion of males or females who played/did not play sport, χ^2^ (3, *N* = 247) = 0.857, *p* = 0.836, with 52.0% of males and 57.5% of females reporting that they did not participate in any sport.

Participants were also asked to indicate the amount of time they spent training and competing in sport (see Table 4). There was a significant difference in the amount of time that males and females spent training and competing in sport, with males spending significantly more time training, *t*_(1394)_ = 3.04, *p* = 0.002, and competing, *t*_(1276)_ = 6.86, *p* < 0.001, in sport overall. When looking across the different year levels, in year 8, there was no significant difference in time spent training, *t*_(688)_ = 0.897, *p* = 0.370, but males spent significantly more time competing in sport, *t*_(629)_ = 3.77, *p* < 0.001. For year 10's, males spent significantly more time both training *t*_(597)_ = 2.60, *p* = 0.010, and competing in sport, *t*_(522)_ = 4.73, *p* < 0.001, than females. For year 12's the same pattern was evident with males who played sport spending significantly more time training, *t*_(105)_ = 2.38, *p* = 0.019, and competing, *t*_(91)_ = 4.84, *p* < 0.001, than females.

**Table 4:** Summary of the amount of time spent playing and competing in a sport.

	**Full Sample**	**Females**	**Males**
	**(*N* = 1461)**	**(*N* = 626)**	**(*N* = 816)**
Variable	Mean (SD)	Mean (SD)	Mean (SD)
*Time spent in sport training*			
Overall (all year levels)	3.51 (2.59)	3.26 (2.62)	3.68 (2.52)
Year level 8	3.19 (2.56)	3.07 (2.69)	3.25 (2.43)
Year level 10	3.82 (2.66)	3.47 (2.64)	4.03 (2.59)
Year level 12	3.86 (2.16)	3.32 (1.93)	4.31 (2.28)
*Time spent competing in sport*			
Overall (all year levels)	2.12 (1.78)	1.71 (1.42)	2.39 (1.95)
Year level 8	2.07 (1.84)	1.74 (1.47)	2.30 (2.07)
Year level 10	2.13 (1.77)	1.69 (1.43)	2.38 (1.84)
Year level 12	2.12 (1.80)	1.53 (0.91)	2.99 (1.71)

## Motivation, Parental Involvement in Sport, and Psychological Factors

Both males and females demonstrated moderate motivation to play sport, with a mean rating of 4.23 out of 7 for all participants (see Table 5). While there was no significant difference across genders in overall motivation to play sport, females reported significantly more introjected motivation (i.e., they were driven more by guilt, shame, or worry) than males, and males reported significantly more external motivation than females to play sport. Not surprisingly, individuals who reported playing at least one sport had greater levels of overall motivation for sport (*M* = 4.30, *SD* = 0.97) than participants who did not play any sport (*M* = 3.53, *SD* = 1.33), *t*_(1482)_ = 6.51, *p* < 0.001. When examining the individual subscales, those who played sport reported higher scores on all subscales, except a motivation. These differences were all significant (all *p*s < 0.001), with the exception of external motivation (*p* = 0.263).

**Table 5:** Summary of sport motivation and ratings of parental involvement in sport for participants who play sport.

	**All**	**Females**	**Males**	***p***
	**(*N* = 1462)**	**(*N* = 626)**	**(*N* = 816)**	
	**Mean (SD)**	**Mean (SD)**	**Mean (SD)**	
*Sport Motivation*				
Total	4.23 (1.03)	4.24 (0.95)	4.23 (1.08)	0.833
Intrinsic motivation	5.27 (1.47)	5.36 (1.44)	5.22 (1.48)	0.065
Integrated motivation	4.76 (1.65)	4.75 (1.62)	4.79 (1.65)	0.715
Identified motivation	5.19 (1.59)	5.28 (1.56)	5.14 (1.59)	0.095
Introjected motivation	4.52 (1.59)	4.68 (1.55)	4.40 (1.61)	<0.001
External motivation	3.08 (1.63)	2.89 (1.59)	3.22 (1.63)	<0.001
Amotivated	2.56 (1.68)	2.49 (1.66)	2.59 (1.69)	0.289
*Parental Involvement in Sport* ^*a*^				
Mum/Caregiver 1				
Directive behaviour	0.02 (0.61)	−0.03 (0.61)	0.05 (0.61)	0.016
Praise and understanding	−0.04 (0.67)	0.03 (0.69)	−0.10 (0.64)	<0.001
Dad/Caregiver 2				
Directive behaviour	−0.12 (0.64)	−0.12 (0.69)	−0.12 (0.60)	0.974
Praise and understanding	0.01 (0.70)	0.11 (0.74)	−0.06 (0.67)	<0.001

a *discrepancy score between perceived and desired behaviour*.

Overall, young people, both male and female, were relatively happy with the amount of support they received from their parents/caregivers for their sport participation, with all scores close to zero for this scale. However, there were differences between males and females in relation to perceived parental support. From Table 5 it can be seen that females desired less directive behaviour from their mother-figures and more praise from both their mother and father-figures than males. There was no relationship between the amount of time spent training or competing in sport and satisfaction with parental involvement (all *p*s > 0.05).

Psychological factors examined included resilience, self-esteem, and body appreciation. There were no differences between those who played sport and those who did not on self-esteem, *t*_(2087)_ = 0.063, *p* = 0.526. However, there were significant differences on resilience, *t*_(2114)_ = 5.78, *p* < 0.001, and body appreciation, *t*_(2081)_ = 7.24, *p* < 0.001. Participants who played sport had higher levels of both resilience (*M* = 3.38, *SD* = 0.66) and body appreciation (*M* = 3.69, *SD* = 0.94) compared to participants who did not play sport (resilience: *M* = 3.21, *SD* = 0.68; body appreciation: *M* = 3.35, *SD* = 1.05). The same pattern of results existed for both males and females (*p* < 0.01).

## Phase 2 Results

### Phase 2 Participant Breakdown

In Phase 2 (focus groups and individual interviews), participants were 21 high-school students, 11 teachers and 5 parents of high-school students. For high-school students across Adelaide schools, a total of four focus groups were conducted, with an average length of 22 mins. For teachers, a total of two focus groups were conducted across two schools, with an average length of 60 mins. For parents, a total of two focus groups were conducted across two schools, with an average length of 44 mins. All of these focus groups and individual interviews were designed to build on the data collected in the surveys and provide illumination surrounding key the issues to emerge within.

## Themes

### Theme 1: Social and Developmental Forces

A delicate combination of friendship and the development of sports skills were central forces for the promotion of sampling sport and continuing a sporting pathway for both boys and girls. However, it was the creation of friendships outside of school that was particularly important for those girls who sought to join a new sport or sporting club. Parents also noted the important role of friendship for ongoing participation in sport during the teenage years particularly with respect to team sports. We found that the club atmosphere was a contributing factor in fostering enjoyment and a setting for developing physical skills and competencies. For many girls, this involved training with their male counterparts to develop skills in Australian football, which is a burgeoning female sport but still a largely masculinised domain. This is in-keeping with other research which showed that when girls feel supported, they are less likely to feel self-conscious while playing sport (Zarrett et al., 2020). The organic blend of social and developmental advancement is a critical factor for supporting girls to sustain their participation. One girl detailed:

Yeah, so I love either being in a team or individual kind of environment. I love the—love the competition as well. So it's just enjoyable being with others. I love also being fit as well. So that team environment, that's always just been a love. (Sophie, year 10, FG 4)

In addition to friendship formation and maintenance, a big indicator of continuation in sport for many of the girls surrounded the security of being socially connected through sport. While peer influence was found to be a strong motivator for initiating participation, it also comprised an important antecedent for transitioning out of one sport and into another. A female year 10 participant in a focus group claimed:

Sometimes your friends can influence whether you want to stay or whether you want to try something else because they might want to do some other sport and you might want to not be by yourself doing the same sport, so you might want to join them or maybe other things you want to do, like other sports or other activities in general that might happen on the same night when you might have training for that sport, or games. So that might give you a reason not to do that sport anymore as well. (Amy, year 10, FG 3)

A significant social factor that emerged was a reduction in the number of sports when adolescents were entering secondary school. Lifestyle priorities was a considerable factor in the reduction of sports as the student were entering year 10 and year 12. Lifestyle priorities consisted mainly of time management between school homework, employment, and school exams. Adolescents reported that “achieving a balance between school and participating in organised sport” was challenging. However, there were a few cases in relation to sport and school homework in that these students found sport as a stress reliever during the school studies. Dual commitments involving sport and academic commitments, nonetheless, remains a key challenge. Teachers consistently witness the juggling of dual commitments by their students. A participant stated:

Yeah, definitely. It's like one of the main reasons I don't play sport because you've got all of these assignments due and all this homework that you need to do and then just a million other things (Josh, year 10, FG 4)

The role of high-quality sports coaches in keeping adolescent girls interested and motivated in organised sport was evident from adolescent participants within this research. Sport enjoyment and skill development was primarily attributed to the quality of community coaching in this study. The development of quality sport coaches was also seen as a priority by teachers and parents in relation to developing a retention strategy surrounding girls and young women. There was also a strong view towards promoting communication and strong relationships between parents and coaches to create a more meaningful and influential impact on girls' attitudes towards sustained sport involvement. The majority of coaches in community sport undertake their role for free, as a volunteer. Similarly, it is recognised that parents contribute an enormous amount by way of volunteerism as well. Ultimately the role of the coach and the parent is critical in keeping girls involved in sport at the community levels (Zarrett et al., 2020). There needs to be acknowledgement of the effort of the coaches and parents in community sport as well as the need for the development of ongoing lines of communication. This fact was not lost on a teacher who stated that:

It would be great if it [parent and coaches involvement] could be improved, but I wouldn't be wanting to put big expectations on them because I think it would die. These people are already giving up a lot of their time and energy. Like I don”t think we give these coaches and parents enough cred personally. But it can be hard. I just have to coordinate some emails and texts and things like that and then you get families—like one family abused the crap out of our coach. (Stuart, School teacher, FG 2).

### Theme 2: Year 12 and the Cost of Sport: Time and Finance

*Time cost:* There are many competing issues to attend with as a young person involved in year 12. There are expectations placed upon year 12 students to “do well” and attain a certain arbitrary ATAR score (Australian Tertiary Admission Rank) that will supposedly “set them up for life”. Parents, teachers, school and individual expectations are all a component of this as many young people have built this period up as the crescendo of their schooling existence. As a consequence, a number of seemingly extraneous activities beyond education in the quest for attaining the desired and expected ATAR fall by the wayside. One of those activities, particularly for girls and young women is their engagement with sport. The year 12 girls in this research identified that time constraints were a major factor in limiting their participation in sport, and physical activity more broadly. There was a perceived underlying tone that it was “just for one year.” However, this year-long hiatus can often be the catalyst to a longer abstention from sport and physical activity, which can lead to many years of non-participation, thereby making it incredibly difficult to return to sport for fear of intimidation, failure or the fact that “life has moved on.” In terms of time costs five year 12 students claimed:

(i) I sort of notice the difference when I came to high-school as well. I started doing a lot of sport in primary school—so soccer, cricket and swimming—then as I started going through high-school, I couldn't do it. (Steph, year 12 FG)
(ii) This year I've had to put all of my sports off. So, I've put them all off in terms of work and school—because they collide. And I found there was no time—I can't do both.
(iii) The last sport I ever did was tennis. And say I was doing Saturday's game and then I was training throughout the week. Trainings would collide with if I needed to study or something like that. And also, I've got work shifts usually on a weekend. And sometimes during the week as well. And on a Saturday, that's when the game is. And that also collided. So, I was unable to go to numerous games, which just—I had to kind of pull out. (Sarah, year 12 FG)
(iv) I guess with schoolwork over the years it was consistent to how much effort I put in. So, say I was doing what I need to get an A in year10 was OK, but if I didn't want to achieve the highest kind of grade I'd put a—like a C grade sort of effort. In year 12 I'd be putting much more time into studying to get an A. (Emma, year 12 FG)
(v) Oh, I got a job this year, so it was really hard trying to tackle this year was just super tough and the job as well. So, I sacrificed stopping sports so I could focus on those 2 things. (Annika, year 12 FG)

Clearly, year 12 students feel competing interests in their final year of secondary school as they navigate through study in addition to taking on the responsibilities of early adulthood, including financial independence, and moving away from parental support. Thereby, less emphasis is on sporting commitments given the, often-regimented, training times that do not necessarily fit into the adhoc times required to work, study and complete assignments. For girls, this seemingly short break from sport can continue upon the completion of high school. One girl stated:

I think there is just a lot of pressure to do well in school, to get your assignments done, get everything done, get a job. Thinking more long-term, what do I need to do to get—to be able to do what I want in the future? And then you also have things that you're also personally interested in that aren't sports related. So, then all of these other things that you just want to do. (so it is easy to drop the commitment of sport). (Sarah, year 12 FG)

*Financial Cost:* As identified above, there is a time cost associated with sport, however financial costs also can negatively impact participation. Although boys may also face financial constraints, they appear to provide an additional “hurdle” that can dissuade girls and young women from sports. Additionally, given that sports are often perceived as rite of passage into manhood for boys and therefore can be viewed as culturally imperative by parents for boys, there may be potentially less emphasis placed on girls' participation, and the ability to “find money” to pay for sports participation may be a higher priority for boys than girls. This ties into the narrative surrounding the importance of education for girls more so than sport. Regardless, the financial cost associated with sports participation is increasing due to a number of contemporary factors including equipment, venue hire and maintenance, legal fees and so forth, which are all built into the cost of memberships. Financial costs can be prohibitive for many families. The three comments by participants reflect these claims:

(i) There's a lot of sporting opportunities. But they're very expensive. And a lot of people can't undertake it because of price, because I know that a few schools in Adelaide that focus on tennis. I know a lot about tennis. But tennis schools are very expensive schools to go to. And say if you're a family that doesn't have that amount of money it's hard to go for that. And also, tennis coaching I used to do, and that's very expensive as well. So, there are a lot of pathways to do it but the expense involved is pretty high. So, some people may not go down that path because of the cost. (Emma, year 12 FG)
(ii) I think a big one is the cost of many sports. I know when I was doing tennis a big reason why I stopped was the cost. To become actually good at it you need all the coaching, and that's expensive plus all the enrolments. And I think it's just really heavy. That's why a lot of people would also quit a lot of sports as well. Just because the cost of it—the cost is very high. (Sarah, year 12 FG)
(iii) And then there is the time and financial cost of transport to sport. I mean there's trains everywhere and trams. Even before I had my licence getting around Adelaide was quite easy and quite good. But sometimes the bus wouldn't be on time. (Jade, year 12 FG)

Irrespective of gender, financial costs associated with sport participation are burdensome. However, similar to the issues associated with time constraints on girls it could be argued that a hiatus of sport participation due to any factor could play a significant role in determining long term participation in that sport, or other sports more broadly. Additionally, if there is strong desire to continue the sport despite financial costs, the time spent earning money to finance the sport participation is ultimately likely to impact sport participation, particularly training and competition times.

### Theme 3: Positive Aspects of Sport for Girls

Although negative impacts of girls' involvement in sports are often discussed, it should be noted that participants discussed a number of positive factors from their sporting participation. Some of the girls identified the importance of staying involved in sport for a range of reasons. These included, fun, friendship and empowerment, which is an important perspective to maintain at an age where girls' sport dropout is high. Throughout the focus groups the girls articulated the meaning of sport to them through detailed conversations. The following dialogue from two girls surrounding the important aspects of sport highlights this claim when they talk about empowerment through sport. However, it is clear that they are feeling empowered by way of the strength that they have attained through the conditioning component of rowing. Indeed, strength has not been an historically major component of many women's sport. A number of women's sports have previously placed emphasis on “grace,” skill and aesthetics. Whereas, in contemporary times, there is an increasing emphasis on women to be strong. The following dialogue is representative of the types of discussion held throughout the focus groups with girls.

Interviewer: So, what are some of the important things about sport?
Sophie: I mean, it's fun.
Jade: It's empowering.
Interviewer: Okay.
Jade: I feel strong when I'm at rowing training, and I spend time with various people.
Interviewer: So, can you explain that?
Sophie: It's enjoyable.
Interviewer: And when you say empowering, what does that mean?
Jade: Like when, say, if we're doing a circuit, if you do something really well, and you just—you feel stronger, and you know that you're improving, because you're doing these things; and with improving in that area, you can improve in other areas of your life.

Importantly, winning and competition was also a significant positive component in the girls' engagement in sport. For a number of the girls, it was a defining factor in terms of why they chose to play certain levels of sport. It was clear that competing in school sports was not as competitive as competing in club or community-based sport, which is emblematic of the sporting structure within Australia. The following dialogue highlights this.

Interviewer: Is there is a difference between playing in clubs compared to school sport?
Kyra: I don't play any school sports.
Ella: Yeah, I prefer out of school sports. (club/community sports)
Interviewer: Why is that?
Ella: I feel like it's just more competitive out of school—I don't know. That's just me.
Kyra: I play school footy and it's very different from club. School footy is more “like everyone is there to have fun and learn a bit,” and that's for most sports. I think that school sport should just be for people that want to try something new and so when there's—I don't know there's really competitive people sometimes they find it difficult—find at different levels I guess of competition. Love the competition, competitiveness and winning.
Interviewer: What do you guys find about sport?
Alyssa: I like the competition and like winning.
Ella: Yeah, winning is pretty good.
Interviewer: So, what motivates you to participate in sport? What else do you sort of find-
Ella: Winning.
Interviewer: Is that about it, anything else?
Ella: Personal results for yourself as well—having goals for yourself that you want to achieve.

### Theme 4: The Rise of Australian Football for Women (AFLW)

While Australian football is a highly masculinised sport within Australian culture, it was also seen as a site of social support for girls. It is a game that requires strength, speed, power and skill. It is also a game with a large number of physical injuries through heavy collisions and general attrition. There have been various football leagues throughout the country that have been in existence beyond 100 years, while the establishment of the National league (Australian Football League; AFL) occurred in the 1990s evolving form the Victorian League dating back to the 1800s. However, in 2016 the AFL women's league (AFLW) was created. There have been widespread plaudits for such an initiative through Australian society and it has challenged the traditional Australian feminised sport of netball in numerous ways through its far more openness to embrace change as well as the competitive and combative nature of the sport. The sport can be seen as a talisman for the change that is seemingly occurring with women's sports more broadly, particularly with respect to engagement in more combative, archetypal masculine sports such as Australian football and rugby. The following discussion highlights the excitement and enthusiasm around AFLW for a number of the girls.

Interviewer: So, tell me about footy (Australian football)?
Kyra: I'm usually in the midfield and, I don't know, I guess the club I'm at is really supportive and everyone is really close. It's like a close group and I just really enjoy being around the people I'm with in the team and so it makes it fun pretty much.
Interviewer: Is that something that you guys enjoy about like if you wanted to be part of sport you need to have, as you say “the right people” around you?
Ella: Yeah.
Alyssa: It makes it more enjoyable if you are doing sport with close people that are you friends with it makes it ‘funner' and you look forward to it—yeah just better.

AFLW has been a significant revelation in Australia with respect to female participation in sport. To be more specific it has provided girls and women with an opportunity to play a sport that has been largely dominated by men. The combative nature of the sport has allowed females to engage in a legitimised heavy contact ball sport. Soccer, basketball and cricket were arguably the closest sports girls and women could play with similar strength, power and sporting prowess. The problem at this point in time is that most of the girls and women playing AFLW has been at the expense of attrition in alternative sports with many “code hopping” from their previous sport to AFLW. The other problem is that the infrastructure that surrounds the sport for girls and women has not been adequately established and could be a problem in the future if it is not addressed. The teachers of the girls at school were quick to identify this fact as they have seen other sports come and go, while the traditional gendered sports (e.g., netball) have ultimately endured. The following conversation with a teacher presents this:

Kate: Yeah, so our girls. With the amateur football clubs that are taking on the girls, they're taking on from under 10 level, and they're really driving it, and the males in the clubs are embracing it, and it's got to a point now, as an example, Hayville, there's more junior girls playing than junior boys, and what's happening now is these clubs are finding that they can't facilitate or cater for the girls, and they're going to have to now renovate their buildings to include girls, females change rooms, find space for them to actually train, because they haven't got enough oval space, and they're trying to find a time to get all of the competitions in, because they don't have a schedule program for competitions. And also umpires, they haven't got enough umpires. So, actually in some areas, the girls are starting to take over in junior boy's football.

Interviewer: So, why is that? Why are they moving towards this expansion?
Kate: Money.
Interviewer: Money?
Kate: The clubs are getting money in from the girls. The girls play—like the boys and they get their fees, they're getting recognised through the media—girls, AFL women's football. I mean, girls find that attractive, they're finding it an easier pathway, SANFL are recruiting now with girls, as young as under 14s, and they're moving out of other sports, like basketball, netball, and they're gravitating towards AFL.
Mark: I think they can see a pathway though sport, as well.
Lisa: They've got role models up there now too.
Dave: Yeah, they've got role models at top level playing AFLW, and they've got something to aspire to. They can see that as an option there, and it's got the option to do it, as a regular team. So, it's taken off. And there's been massive promotion for it as well, and they're choosing it.
Lisa: And with the success of the Australian women's rugby team, as well, at the Olympics, I think that's been another positive for the girls to look up to, you know?

Within the context of these claims around AFLW and the perception of the sport being somewhat masculinised and combative there was a chorus of girls suggesting that contemporary females should not be stereotyped in traditional feminised ideological sports. As one teacher aptly suggested:

You don't stereotype girls. It's a simple as that. Like there's—so, now particularly with the AFL coming in, all of a sudden that's shown that you don't have to—you can't—put girls in the box of netball, or softball and stuff like that. It's now a broad spectrum of sports, and you need to allow them to –the eight percent, or whatever it number of girls it is, to go and play football, and the other twenty percent, they go and play netball.

## Discussion

The aim of this study was to examine the intrapersonal, interpersonal, and environmental influences on community sporting pathways for girls and young women in Adelaide, South Australia. We chose to investigate the sporting practises and insights of males of the same age group in order to juxtapose the findings of the girls' and young women's results in order to provide a more comprehensive understanding of girls' and young women's sporting involvement.

The results from the survey indicated that more than 67% of the students that completed the survey were currently involved in at least one organised sport or had been involved in at least one organised sport in the last 12 months. This included 64% (*n* = 626) of all female and 70% (*n* = 816) of male respondents. Consistent with other Australian research examining peak age for youth sport drop out, participation declined with age, before year 10 for girls, and before year 12 for boys (Eime et al., 2020; Zarrett et al., 2020).

The results of the survey suggest that in general, boys play more sport, and compete more, than girls. However, gender differences in sport participation are apparent and rates of sports participation decline with age for both genders. Therefore, within the context of this research and the for the purposes of this paper, it would be important to intervene before year 10 for girls. A recent study by the Youth Sports Trust (2017) of 26,000 girls and boys across England and Northern Ireland displayed a significant difference in girls' attitudes and actual physical activity levels. Their findings indicated that school-aged girls participate in less physical activity and sport than their male counterparts, with girls facing barriers such as low self-confidence, self-consciousness, the academic pressure of school, and severe body image dissatisfaction. The findings of the current study also found that dual commitments such as academic study and sport were difficult to manage. However, those who played sport had significantly higher resilience and body appreciation than those who did not play sport—indicating the importance of focusing on these factors to promote sports participation in those who do not play sport. For enhancing body appreciation among girls, it is important to focus on what the body can do, rather than how it looks. Therefore, while low perceptions of body appreciation are linked with low-to-no sport participation among young people in South Australia, sport may also be an avenue for building and/or maintaining positive self-perceptions and cultivating psychological resilience. This could help to inform broader messages around the benefits of ongoing sport participation.

To advance theoretical understandings of what factors support and inhibit female involvement along the sporting pathway, the qualitative findings suggest that a delicate blend of social and developmental progress comprises a vital source of encouraging initial and ongoing participation. Clubs and organisations seeking to maximise girls' participation rates might therefore reflect on their policies and practises against these criteria to assess if the sport climate and these initial experiences respond to the motivational climate girls prefer. This is strongly supported by other research which indicates that coaches and the sporting clubs themselves help shape the sporting experiences of girls who play sport (Farmer et al., 2018; Zarrett et al., 2020; Eliasson and Johansson, 2021). Our results indicate that the careful advancement of girls' social and development progress is key to continuation along a sporting pathway.

Another challenge to maintaining involvement in or transitioning into a sporting pathway was girls and young people's inability to manage dual academic and sport commitments. This is consistent with other similar research (Elliott et al., 2018). However, our study indicates that while school priorities place pressure on ongoing sport participation, sport was also viewed as a stress reliever among young people. One implication is for government bodies and associated sporting organisations to develop information, support and advice to assist young people to manage dual commitments given the role of sport in reducing perceptions of school-related stress. This requires a reconceptualisation of sport and school commitments as complimentary rather than competing priorities.

Quality coaching was also regarded essential to developing a broader strategy to engage girls and young adults into sporting pathways long- term. However, this was not limited to merely their pedagogical capability. Rather, participants discussed the importance of coaches developing relational skills to improve communication with participants as well as parents and other adults in the sport setting. Perceptions of positive parent and coach relationships are associated with creating a task-focused motivational climate, which are linked with sport continuation (Zarrett et al., 2020). Therefore, our findings provide support for sporting clubs and organisations to develop coach qualities that advance their interpersonal skill and communication as a way of retaining girls in the sporting pathway. This also offers theoretical potential to enhance parent and coach relationships, which may lead to increased volunteerism and organisationalsupport.

There were several strengths of this research including:

- The use of six validated instruments to measure students' opinions and attitudes of their participation in organised sport.
- Surveying nine schools across Adelaide, South Australia, resulting in a significantly large sample size (*N* = 2189) that is representative of the population in Adelaide, South Australia.
- The use of a mixed-methods explanatory research design to assist in the triangulation of findings of the quantitative results and the qualitative results. This assisted in providing a multidimensional perspective of the phenomenon with reliable, rigorous and contextually rich data that can interpreted with some degree of assurance.
- We employed several means of qualitative excellence to ensure methodological rigour including the use of a critical friend, field notes for auditability, intense time in the field, methodological triangulation and sincerity in reporting the findings. These are key markers of excellence in qualitative fieldwork.

Importantly, it is noteworthy that there were also some potential limitations of the study including:

- School recruitment involved convenience sampling methods which may have resulted in attracting schools that have a strong emphasis on sport and the development of elite athletes. Every effort was taken to obtain a randomised sample within a two-stage, stratified, cluster design methodology, from government, catholic and independent school systems representing all socioeconomic tertiles.
- Our method involved surveying the entire school in years 8, 10 and 12 with the use
- of an opt-out approach as a proxy to approximate equal chance of being selected in the sample and obtain a sample that is representative of Adelaide, South Australia.
- Self-report measures were used in the Phase 1 survey to allow for a large sample size of high-school students, however the use of clinician-administered interviews may provide more accurate results.
- Perceived parental involvement in sport in the present study was measured using an adapted version of the Parental Involvement in Sport Questionnaire (PISQ; Lee and MacLean, 1997), to allow assessment of a wide range of sports (e.g., “hockey” changed to “sport”); however future studies should assess the validity of this adapted version.
- Although there was sufficient qualitative depth and richness to satisfy the objectives of the project, another potential limitation was the limited number of parents involved in focus groups. Children's and teachers' perspectives were dominant voices in the data, but parents also hold rich and informative experiences which could have further enhanced, expanded or extended our understanding. Similarly, conducting focus group discussions with coaches would have provided valuable insights into the phenomenon.

Overall, this study provides new insights into the phenomena of pathways to organised sport in the education context and in the wider sporting community for girls, boys and young adults. The findings indicated that gender differences in sport participation are apparent and rates of sports participation decline with age for both genders. We identified a decline in sport participation before year 10 for girls, and before year 12 for boys. Participants who played sport had higher levels of both resilience and body appreciation compared to participants who did not play sport. The qualitative and quantitative data within this research have highlighted several issues that concern sport participation, retention and dropout in metropolitan South Australia. We conclude with seven recommendations that are extrapolated from our data.

## Recommendations

1. State Sporting Policies, Funding Programs and Organisations Should Intensify Focus on Retention Practises That Extend Beyond Promoting Participation and Developing Inclusive Environments. Funding and Policies Aimed at Demonstrable Improvements in Retention Practises Should be Given Equal Importance to Efforts Seeking to Promote and Grow Participation Particularly With Respect to Girls Prior to Year 10.
2. Strategic Investment in Research Is Required to Improve Efficiencies Between School and Community Sport Programs Designed to Promote Girls' Participation and Retention, Develop Talent, and Sustain Longer-Term Involvement of Players, Coaches, Parents and Volunteers.
3. As a key Retention Strategy, Invest in Evidence-Based Resources for Parents, Teachers and Adolescents That Assist Girls and Young Women to Develop Skills and Strategies to Maintain a Dual Commitment Between Academic Study and Organised Sport Participation.
4. Investment in Community and Research-Based Projects That Seek to Enhance Coaches' and Parents' Relational Capabilities and Pedagogic Contributions to Community Sport to Reinforce Long-Term Attitudes and Practises Conducive for Girls' Sport Participation and Retention.
5. To Build Intrinsic Motivation and Positive Perceptions of Competence, Develop an Integrated and Theoretically Informed Approach to Prolong Girls' and Young Women's Diversification (e.g., Sample More Than one Sport) in Sport Into Late Adolescence.
6. Create Multifaceted Support Materials and Programs to Assist Girls to Sustain a Sporting Trajectory Between Year 8 and 10 to Address Sharp Declines in the Sporting Pathway.
7. Develop State-Based Campaign That Promote the Strong Association Between Sport Participation and Higher Body Appreciation and Resilience as a Strategy to Engage More Families and Girls in Community and School-Based Sport.
8. Seek Government and School-Based Solutions to Arrest the Decline in Sport Participation Among Girls in Year 12.

## Data Availability Statement

The original contributions presented in the study are included in the article/supplementary material, further inquiries can be directed to the corresponding author.

## Ethics Statement

The studies involving human participants were reviewed and approved by Flinders University Social and Behavioural Research Ethics Committee. Written informed consent to participate in this study was provided by the participants' legal guardian/next of kin.

## Author's Note

The researchers agreed to name the survey “The Young People in Sport Survey” to ensure that boys and young men were also encouraged to engage in the research.

## Author Contributions

MD and CD worked on the original version. SE then worked on the next version. IP, LL, and J-LP were responsible for the statistics design and analysis. CB and NB collected the data and assisted in the final version of the paper. All of the researchers were involved in the research from the beginning.

## Funding

This paper is based on a research grant received from the Office for Recreation, Sport and Racing, South Australia.

## Conflict of Interest

The authors declare that the research was conducted in the absence of any commercial or financial relationships that could be construed as a potential conflict of interest.

## Publisher's Note

All claims expressed in this article are solely those of the authors and do not necessarily represent those of their affiliated organizations, or those of the publisher, the editors and the reviewers. Any product that may be evaluated in this article, or claim that may be made by its manufacturer, is not guaranteed or endorsed by the publisher.
